# A Novel Adaptive Deformable Model for Automated Optic Disc and Cup Segmentation to Aid Glaucoma Diagnosis

**DOI:** 10.1007/s10916-017-0859-4

**Published:** 2017-12-07

**Authors:** Muhammad Salman Haleem, Liangxiu Han, Jano van Hemert, Baihua Li, Alan Fleming, Louis R. Pasquale, Brian J. Song

**Affiliations:** 1 School of Computing, Mathematics and Digital Technology, Manchester Metropolitan University, Manchester, M1 5GD UK; 2 Optos Plc, Queensferry House, Carnegie Business Campus, Enterprise Way, Dunfermline, Scotland, KY11 8GR UK; 3 Department of Computer Science, Loughborough University, Loughborough, LE11 3TU UK; 4 Department of Ophthalmology, Massachusetts Eye and Ear Infirmary, Harvard Medical School, Boston, MA USA

**Keywords:** Medical image processing and analysis, Machine learning, Computer-aided retinal disease diagnosis, Glaucoma

## Abstract

This paper proposes a novel Adaptive Region-based Edge Smoothing Model (ARESM) for automatic boundary detection of optic disc and cup to aid automatic glaucoma diagnosis. The novelty of our approach consists of two aspects: 1) automatic detection of initial optimum object boundary based on a Region Classification Model (RCM) in a pixel-level multidimensional feature space; 2) an Adaptive Edge Smoothing Update model (AESU) of contour points (e.g. misclassified or irregular points) based on iterative force field calculations with contours obtained from the RCM by minimising energy function (an approach that does not require predefined geometric templates to guide auto-segmentation). Such an approach provides robustness in capturing a range of variations and shapes. We have conducted a comprehensive comparison between our approach and the state-of-the-art existing deformable models and validated it with publicly available datasets. The experimental evaluation shows that the proposed approach significantly outperforms existing methods. The generality of the proposed approach will enable segmentation and detection of other object boundaries and provide added value in the field of medical image processing and analysis.

## Introduction and background

**Fig. 1:**
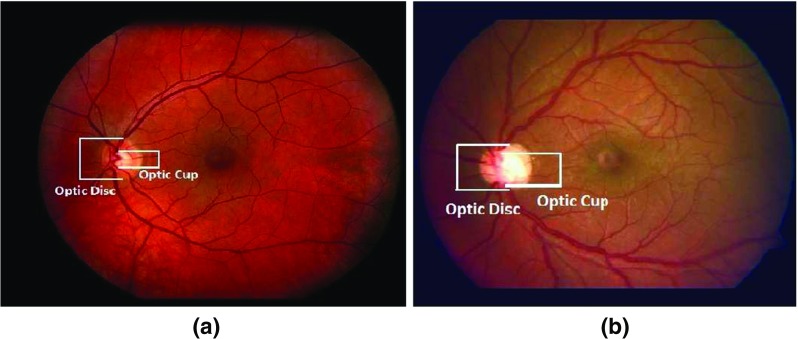
Comparison of CDR in **a** normal image and **b** glaucoma image. The glaucoma image has higher CDR

**Fig. 2:**
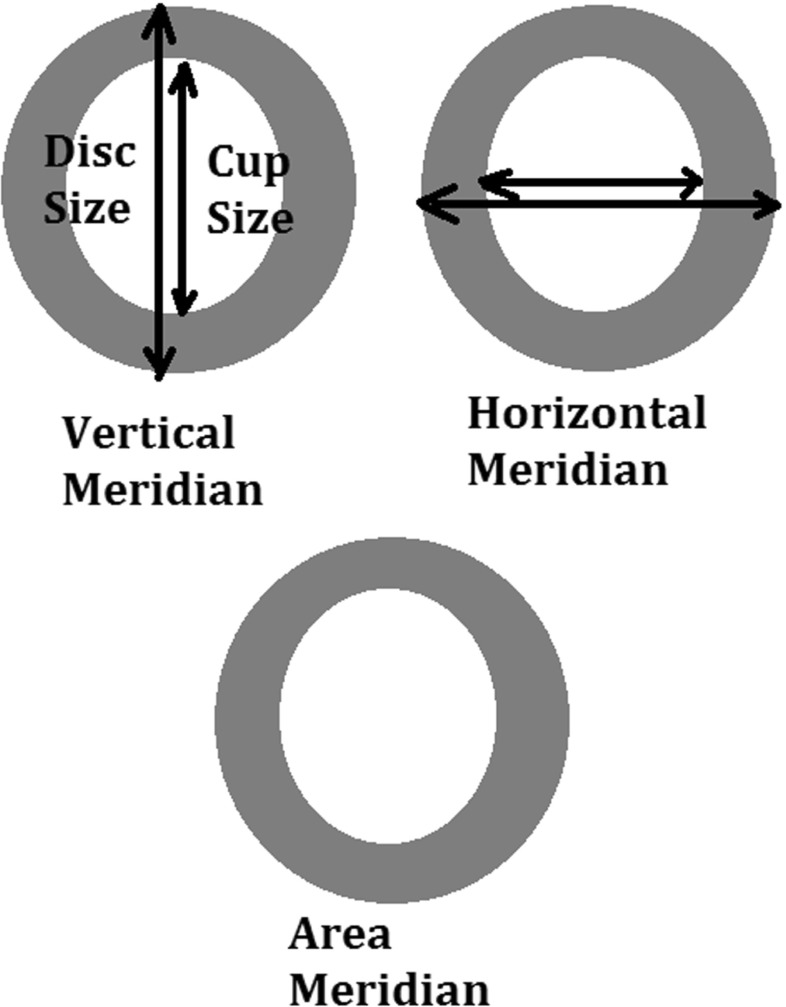
Different meridians of Cup to Disk Ratio (CDR) measurement

Automated boundary detection of optic disc and cup plays an important role for the computer-aided diagnosis of retinal diseases from ophthalmic images. In a typical 2-Dimensional (2D) retinal image, the optic disc is a bright elliptical structure with a cup and surrounding rim tissue. Changes in the shape and depth of the cup or colour of the rim tissue represent signs of optic neuropathology such as glaucoma, which is a leading cause of irreversible blindness. In the case of glaucoma, nerve fibre atrophy is accompanied by erosion of rim tissue, which manifests as cup enlargement. The degree of cupping is quantified clinically as the horizontal, vertical and area Cup-to-Disc Ratio (CDR) (Figs. 1 and 2) [1, 2]. In current clinical practise, the size of the cup relative to the disc is estimated subjectively which is both time-consuming and prone to inter-observer variability [3]. Therefore, accurate automatic boundary detection of optic disc and cup is critical for the diagnosis of glaucoma.

Some research efforts have been made on automatic segmentation of the optic disc or optic cup [4–11]. These efforts can be broadly divided into two categories: non-model-based and model-based approaches. In the non-model-based approaches [12–15] the boundary detection mainly uses some algorithms such as thresholding, morphological operations, pixel clustering etc. However, the identification process of the optic disc boundary had some problems due to obscuration by bridging retinal vessels. The model-based approaches can be classified into shape-based template matching and deformable modelling approaches. The template matching focuses on incorporating the shape of the object and its grey-level appearance in an image by matching optic disc edge with a circular [16, 17] or elliptical shape [18]. But these methods suffer from inaccuracy since they often fails to detect shape irregularity due to the shape variation of an object. The deformable modelling can be further classified into either free-form or parametric deformable models. The free-form deformable models have no global structure of the template, which can freely deform the shape of an object. Typical examples of free-form deformable models include the active contour or ‘snakes’ model (ACM), level-set model [19–21] and Chan-Vese (C-V) level set model [22] (a type of ACM model without edge) and their variations [23–28]. The parametric deformable models involve an offline training process to determine a shape model parameterising diverse shape characteristics, such as Active Shape Model (ASM) [29–31]. The ASM-based approach refers to shape approximation of an object using statistical approaches to learn the boundary shape of the optic disc from a training set [32–34].

The aforementioned deformable models have shown promise for segmentation of the optic disc or cup. However, there are still challenges related to achieving high accuracy in optic disc and cup segmentation. For instance, in some cases, the optic disc might not have a distinct edge due to disc tilting or peripapillary atrophy (PPA) and disc vessels could misguide the segmentation. The ASM does not adequately segment optic discs with PPA [34]. Additionally, since pathological changes may arbitrarily deform the shape of the optic disc and also distort the course of blood vessels, the existing ASM-based approaches fail to accurately extract the object boundary with variation and irregularity and are influenced by blood vessel obscuration. The modified ACM approach as proposed by Xu and colleagues [35] have addressed optic disc segmentation problems due to vasculature occlusion and PPA by adjusting the uncertain cluster points of the contour. Nevertheless, they have also observed optic disc segmentation failures due to retinal atrophy or bright retinal lesions. Furthermore, the method is dependent on initialisation parameters as it relies on local gradient information only. The accuracy of the Chan-Vese (C-V) level set model is dependent on the initialisation parameters as well. Despite the application of gabor filters at different frequencies used after vasculature removal to reduce the PPA occlusion [23], the filtering parameters may need to be modified manually for better accuracy. Vasculature removal with morphological filtering can also diminish the optic disc edges which can affect the segmentation accuracy.

To address the limitations above, this research has proposed a novel adaptive deformable model for automatic segmentation of the optic disc and cup capable of capturing shape variation and irregularity. Our approach doesn’t need to fit a predefined template or shape constraints. The major contributions include:

1. A new Region Classification Model (RCM) which identifies the initial optimum contour approximation representing optic disc or cup boundary between inside and outside a Region of Interest (ROI) based on pixel-wise classification in a multidimensional feature space (with features extracted on the pixel level). The multidimensional feature space represents the local textural, gradient and frequency based information which is used as input for training a backpropagation Neural Networks classification model of an optimum contour approximation.
2. A new Adaptive Edge Smoothing Update model (AESU) for contour regularisation and smoothing update based on iterative force field calculations with any contour obtained from the RCM.

The rest of this paper is organised as follows: “The proposed adaptive region-based edge smoothing deformable model” details the proposed model; “Experimental evaluation” describes datasets used, evaluation metrics and experimental results; “Conclusion” concludes the work and highlights future work.

## The proposed adaptive region-based edge smoothing deformable model

### The rationale

**Fig. 3:**
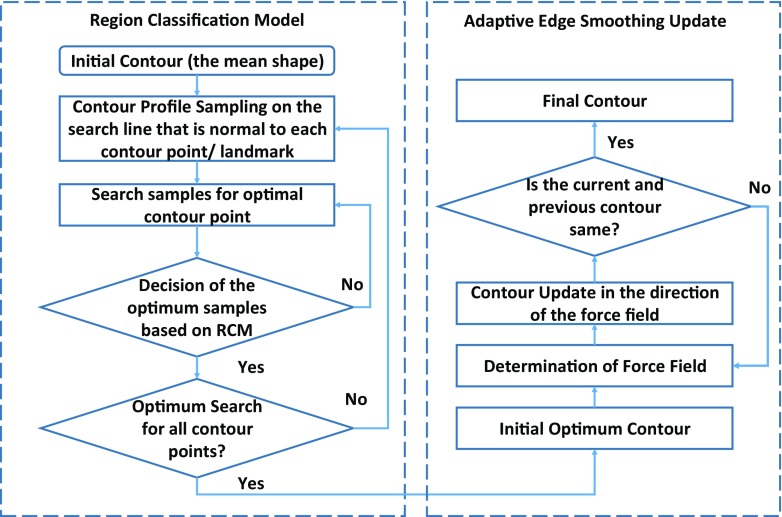
Block diagram of adaptive region-based edge smoothing model

The proposed adaptive region-based edge smoothing model (ARESM) aims to accurately detect the boundary of an object and search optimum contour points in an iterative way. As shown in Fig. 3, the principles of the proposed method mainly include: 1) The Region Classification model (RCM) that generates the initial optimum contour; 2) the Adaptive Edge Smoothing Update (AESU) model that dynamically updates the initial optimum contour obtained from the RCM for accurate detection of the boundary. The segmentation focuses on the pixel-based image representation in a multidimensional feature space by applying Gaussian [36], Dyadic gaussian [37] and Gabor filters [38] at different scales on both the red and green image channels to make use of local information and provide power in capturing shape variation and irregularity.

Different from the existing approaches, the proposed method extracts the feature information at the pixel-level, identifies the initial optimum contour based on regional classification, and dynamically updates the contour without requiring a predefined template such as a circular or elliptical shape. The model uses only once the mean of shapes in the training set as an initial parameter. The model will then search the optimum contour points based on the RCM classification, which is not necessarily identical with the landmarks of the mean shape and may dynamically change during the search. The shape irregularities and misclassified contour points are then dynamically updated and smoothed by the AESU model through the minimisation of energy function based on the force field direction. The proposed approach can capture shape variations and irregularity very well, for example, in the presence of PPA or vasculature occlusion.

### Pixel-based image representation in multidimensional feature space

To provide robustness against variations found in and around the Region of Interest (ROI), we consider features on the pixel-level to fully take advantage of local image information. Each pixel of an image belongs to a homogenous region corresponding to an object or part of an object. We have generated a multidimensional feature map by applying the Gaussian, Dyadic Gaussian and Gabor filters at different scales on both the red and green channel of an image. These features represent the gradient, texture and the frequency based information. The details of these features have been based on our previous work [40] and are summarised in Table 1. This is in contrast to the previous pixel-based approaches which focus on either the red channel or its gradient. The advantage is to extract the representative pixel-level features of an image and accurately classify between the region inside and outside the boundary of an object (e.g. optic disc or cup). For the boundary of the optic disc, its region outside includes the retinal area and the PPA and the region inside includes Optic Nerve Head (ONH) rim and optic cup. For the boundary of the optic cup, we extract features and classify between the region inside of the optic cup and the ONH rim. The retinal vasculature area is also converged inside the ONH area and obscures both the optic disc and the optic cup margin. To investigate the influence of vasculature, we have calculated the feature map under two situations: a) with vasculature removal and b) without vasculature removal. The comparison of the classification power of the features with and without vasculature removal has been described in the experimental section.

**Table 1:** Summary of Gaussian and Gaussian derived features

Feature name	Equation	Feature name	Equation
Gaussian filter	$\mathcal {N}(\sigma ,i_{x},j_{y}) = \frac {1}{2\pi \sigma ^{2}}e^{-\frac {{i_{x}}^{2} + {j_{y}}^{2}}{2\sigma ^{2}}}$	Gamma-normalised derivative	$L_{pp,\gamma -norm} = \frac {\sigma ^{\gamma }}{2}(\mathcal {N}_{xx}+\mathcal {N}_{yy}- \sqrt {{(\mathcal {N}_{xx}- \mathcal {N}_{yy})}^{2}+ 4{\mathcal {N}_{xy}}^{2}})$ ($\frac {\sigma ^{\gamma }}{2}$ is normalisation factor with $\gamma =\frac {3}{2}$)
Dyadic Gaussian	$I_{mn} = \frac {(R+G)}{2}$ *Y*_*rg*_ = *R* + *G* − 2|*R* − *G*|		$L_{qq,\gamma -norm} = \frac {\sigma ^{\gamma }}{2}(\mathcal {N}_{yy}+ \mathcal {N}_{yy} + \sqrt {{(\mathcal {N}_{xx}-\mathcal {N}_{yy})}^{2}+ 4{\mathcal {N}_{xy}}^{2}})$
	*I*_*mn*_(*c*,*s*) = |*I*_*mn*_(*c*) − *I* *n* *t* *e* *r* *p*_*s*−*c*_ *I*_*mn*_(*s*)|	Differential Geometric Edge Definition	$L_{uu} = \mathcal {N}_{xx}+\mathcal {N}_{yy}$
	*R* *G*(*c*,*s*) = |(*R*(*c*) − *G*(*c*))		$L_{u,u} = {\mathcal {N}_{x}}^{2}+{\mathcal {N}_{y}}^{2}$
	− *I* *n* *t* *e* *r* *p*_*s*−*c*_(*R*(*s*) − *G*(*s*))|		
	*Y*_*rg*_(*c*,*s*) = |(*Y*_*rg*_(*c*)) − *I* *n* *t* *e* *r* *p*_*s*−*c*_(*Y*_*rg*_(*s*))|		$L_{uv} = {\mathcal {N}_{x}}^{2}\mathcal {N}_{xx} + 2\mathcal {N}_{xy}\mathcal {N}_{x} \mathcal {N}_{y}+{\mathcal {N}_{y}}^{2}\mathcal {N}_{yy} $(*u*,*v*) are local coordinate system [39]
Gabor	$Gb(x,y,\gamma ,\lambda ,\sigma ,\theta ) = \exp (-\frac {1}{2}(\frac {{\hat {x}^{2}}}{\sigma ^{2}}+\frac {\hat {y}^{2}\gamma ^{2}}{\sigma ^{2}})* $	Difference of	${\Gamma }_{\sigma _{1},\sigma _{2}}(x,y)$ = $\frac {1}{\sigma _{1} \sqrt {2\pi }}e^{-\frac {x^{2}+y^{2}}{2{\sigma _{1}^{2}}}}$-$\frac {1}{\sigma _{2}\sqrt {2\pi }}e^{-\frac {x^{2}+y^{2}}{2{\sigma _{2}^{2}}}}$
	$\exp (\frac {i2\pi x}{\lambda })$	Gaussian (DOG)	
	$\hat {x} = x cos\theta + y sin\theta \qquad \hat {y} = y cos\theta - x sin\theta $		

*i*_*x*_ and *j*_*y*_ are the pixel coordinates of the filter. $\mathcal {N}(\sigma )$ is Gaussian filter, $\mathcal {N}_{x}(\sigma )$ and $\mathcal {N}_{y}(\sigma )$ are first order derivatives and $\mathcal {N}_{xx}(\sigma )$, $\mathcal {N}_{xy}(\sigma )$ and $\mathcal {N}_{yy}(\sigma )$ are second order derivatives of Gaussian filter in both horizontal(x) and vertical(y) directions. *I*_*mn*_ and *Y*_*rg*_ are mean and mixed reponses of both Red(*R*) and Green(*G*) channels respectively at centre levels *c* and surround levels *s* of the spatial scales, *s* = *c* + *d*. *I* *n* *t* *e* *r* *p*_*s*−*c*_ is the interpolation to *s* − *c* level. *σ*, *σ*_1_, *σ*_2_ = 2, 4, 8, 16. *c*, *d* = [2, 3, 4]. $\gamma =[\frac {1}{3},\frac {1}{2},1,2,3]$, $\lambda =[\frac {1}{3},\frac {1}{2},1,2,3]$, *𝜃* = [0^∘^, 45^∘^, 90^∘^, 135^∘^]

**Fig. 4:**
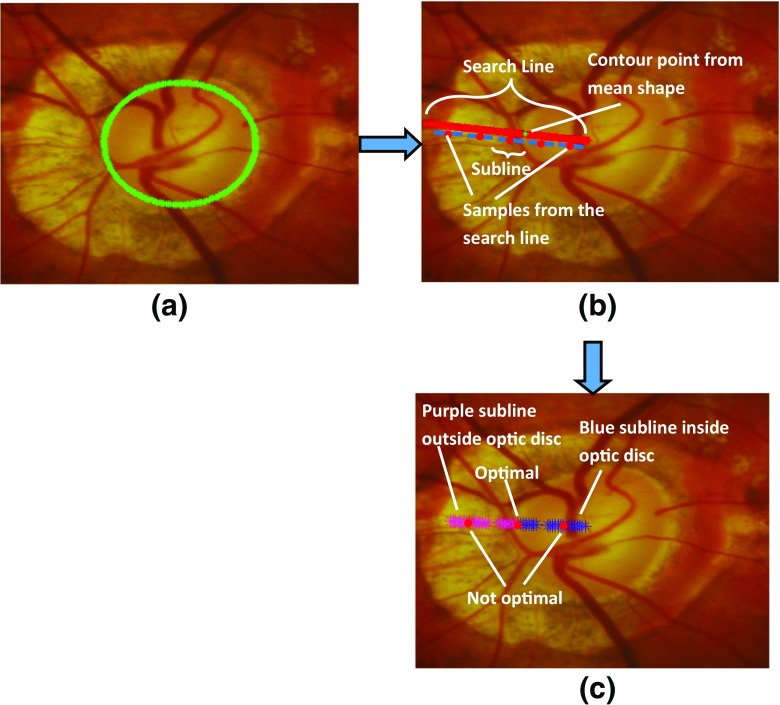
Contour profile sampling steps to determine the disc boundary in this challenging optic nerve photo due to extensive PPA with **a** Mean shape initialisation, **b** Sampling the search line (red) normal to the contour point. Each sample on the search line has its subline samples. **c** Determination of optimal sample on the search line

### Feature extraction and selection

The feature space after applying the different filters above on the pixel-level is of high dimensionality. To obtain the most relevant features for the respective classification, we have adopted iterative sequential maximisation of task performance (also called the ‘wrapper feature selection’ [41]) in which initially the data is divided into *k* folds (in our case *k* = 5). Then the first feature is selected which has maximum mean classification performance across the folds. During subsequent iterations, the features together with previously selected features resulting in the highest mean classification performance are selected. This process continues until there is little or no maximisation (less than 0.01) towards classification performance. For the quantification of classification performance, we have certain performance measures such as Area Under the Curve (AUC), Linear Discriminant Analysis (LDA) accuracy and Quadratic Discriminant Analysis (QDA) accuracy [42].

### Region classification model for initial optimum contour approximation

The RCM consists of two main steps: 1) Initialisation of shape or contour profile; 2) Contour profile optimisation.

#### Initialisation of shape or contour profile

The RCM requires an initial shape profile which is then dynamically updated. In our approach, we use the mean of the shape from the training set as an initial input. It is worth noting that the initial optimum contour profile is not necessarily identical with the mean shape and can dynamically change during the search. To obtain the initial shape (the mean of the shapes), we have used Procrustes alignment [43]. The shape alignment aims to transform all training set shapes into a common coordinate frame. Given an object shape, described by *n* points, referred to as landmark points which can be obtained from the training sample images manually annotated by clinicians. Each shape can be described as a vector of *n* coordinate pairs having 2*n* elements as mentioned below:

1 $$ \textbf{v}=[(x_{1},y_{1}),(x_{2},y_{2}),........,x_{n},y_{n}].  $$

The shape alignment can be achieved by translating the shapes to the origin. Initially, the centroid of each shape is calculated as the average position of all its landmarks, then the shapes are translated to its local origin by subtracting its centroid. The centroid of the shape can be represented as:

2 $$ \textbf{v}_{cent}=(\bar{x},\bar{y})=(\frac{1}{n}\sum\limits_{j = 1}^{n} x_{j},\frac{1}{n}\sum\limits_{j = 1}^{n} y_{j}).  $$

and the shapes are translated to the origin as:

3 $$ \textbf{v}_{trans}=\textbf{v}-\textbf{v}_{cent}  $$

where, $\textbf {v}_{trans}=\textbf {v}-(\frac {1}{n}{\sum }_{j = 1}^{n} x_{j},\frac {1}{n}{\sum }_{j = 1}^{n} y_{j})$ is the shape translated to local origin after subtracting its centroid. We obtain the mean of *N* aligned shapes present in the training set as:

4 $$ \bar{X}=\frac{1}{N}\sum\limits_{i = 1}^{N} \textbf{v}^{i}_{trans}.  $$

#### Contour profile optimisation

After obtaining the mean shape from the training set (Fig. 4a), we determine a preliminary optimum contour around this initial region. The essence is to find the best contour points around the initial region (the mean shape). We have developed a classification model based on a backpropagation artificial neural network (ANN) [42] that creates a classification of pixels as likely belonging to the optic disc or to background. We have performed a search to find an initial outline of the optic disc. A search is made along the *n*_*p*_ samples of each radial line extending from the centre to the edge of the image, as shown in Fig. 4b. A sliding window of length 2*m*+ 1, where *m*= 7 was defined empirically, traversed the radial line (Algorithm 1), to find the optimum transition from pixels which were highly likely to be optic disc to pixels highly likely to be background, as determined by the ANN (Fig. 4c). The optimum transition is where Eq. 5 is minimised. Considering that whilst training the classification model (i.e. backpropagation ANN classification model in our case), the samples outside the optic disc are labelled as 0 and samples inside the optic disc region are labelled as 1, we have the equation to determine optimum position of the contour point:

5 $$ pf(j) = (\frac{{\sum}_{k = 1}^{m}net_{j}(g_{k})}{m}+ 1-\frac{{\sum}_{k=m + 2}^{2m + 1}net_{j}(g_{k})}{m}) $$

where *g* is the subline of 2*m* + 1 subsamples and *g*_*k*_ is the subsample of the subline *g*. The value of *j* corresponds to the sample at the search line. The factor $\frac {{\sum }_{k = 1}^{m}net_{j}(g_{k})}{m}$ is supposed to be a group of subsamples from outside of the optic disc and expected to have logistic output near to 0 whereas the factor $\frac {{\sum }_{k=m + 2}^{2m + 1}net_{j}(g_{k})}{m}$ is expected vice versa. *n* *e* *t*_*j*_(*g*) is the neural weight of the respective profile at sample *j* of the search line which is obtained based on ANN with the backpropagation algorithm. The ANN is composed of an input layer, a hidden layer and an output layer. The number of units in the input layer are equal to the number of selected features. The hidden layer is the weighted combination of units of the input layer. In the backpropagation algorithm, the weights of the hidden and the output layer are adjusted according to the error between the expected output and the actual output. The logistic output of the ANN is given with the following sigmoid function as:

6 $$ net = \frac{1}{1+exp^{-w_{o} - {\sum}_{h} w_{h} (w_{o} + {\sum}_{i}w_{i}x_{i})}} $$

The variables *w*_*i*_ and *w*_*h*_ represent the weights to the input of output layer and hidden layer respectively, *w*_*o*_ is the bias input value, whereas *x*_*i*_ are samples from the selected feature maps. The optimisation algorithm is shown in Algorithm 1. 
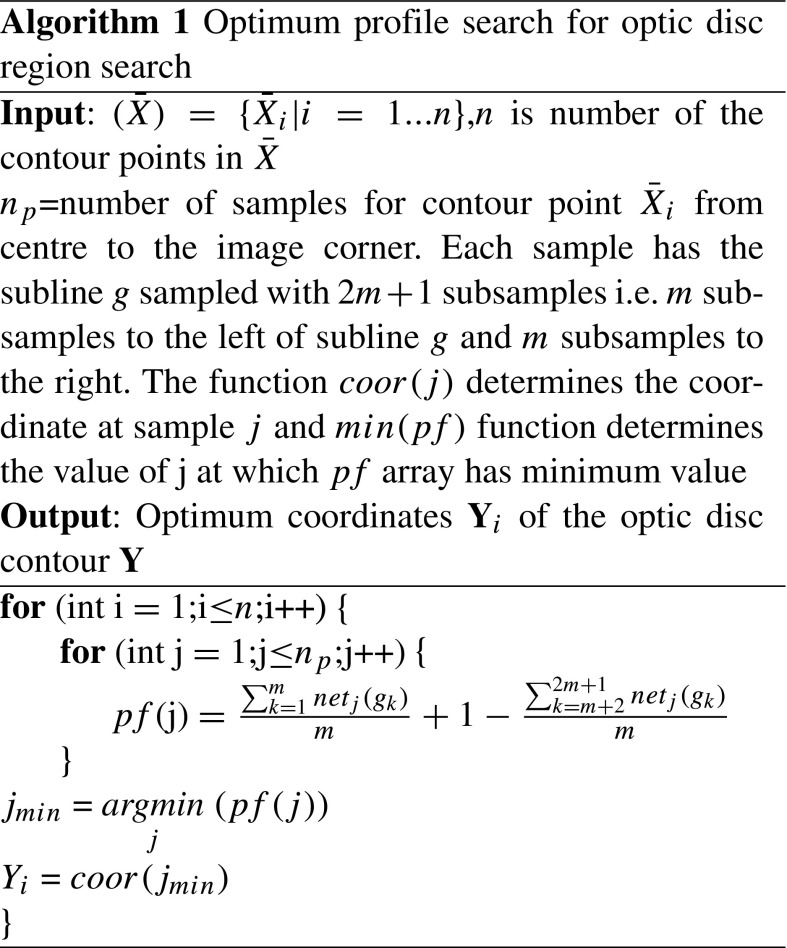


**Fig. 5:**
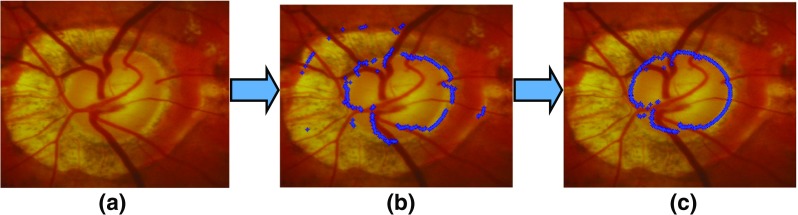
An example of optic disc segmentation by our proposed algorithm with **a** example image from a disc with extensive cupping and peripapillary atrophy , **b** output after optic disc region search by the RCM and **c** output after optic disc shape edge update

The RCM proves to be accurate and shows most of the contour points have been classified correctly as the optic disc boundary (Fig. 5a). However, some of the contour points can be misclassified on disc with extensive cupping and PPA as shown in Fig. 5b, c. Therefore, in order to overcome the classification error, we have developed an adaptive edge smoothing scheme to perform the contour update as explained in subsequent sections.

### Adaptive edge smoothing update

After the determination of tentative optimum contour **Y** from the RCM above, the shape can still be irregular due to misclassification as shown in Fig. 5b. There have been several techniques such as least square regression or circular or ellipse fitting to approximate the shape of the contour [16–18]. However, these methods have limited capability in detecting shape irregularity. The ASM shape approximation approach tends to keep the shape consistent with the mean shape of the training set (the shape parameter is static) and can not dynamically adapt to the actual boundary of the object (as each object has its variability). Different from existing methods, we propose an adaptive edge smoothing update (AESU) model to dynamically update the contour profile generated from the RCM. The AESU is an iterative model which is updated according to the force field direction in every step by a minimising energy function of grey-level image *I* as $\epsilon = \int \int ({u_{x}^{2}}+{u_{y}^{2}}+{v_{x}^{2}}+{v_{y}^{2}})+||\nabla I||^{2}||\textbf {F}-\nabla I||^{2}dxdy$ computed from Gradient Vector Flow (GVF) [44]. For minimisation of energy function, we need to determine force field **F**(*x*,*y*) = [*u*(*x*,*y*),*v*(*x*,*y*)] at pixel (*x*,*y*). The *u*_*x*_, *u*_*y*_, *v*_*x*_ and *v*_*y*_ are force field derivatives along the x and y direction. In contrast to existing approaches such as the Hough transform, we need not assume that the optic disc is either circular or elliptical in shape. The optimum contour **Y** obtained from the RCM can be redefined as:

7 $$ \textbf{Y}{x_{i}}=x_{c}+D_{i}cos\theta_{i} \qquad \textbf{Y}{y_{i}}=y_{c}+D_{i}sin\theta_{i} $$

where *x*_*c*_ and *y*_*c*_ are the centre of the contour **Y**. In the first step of the iteration, they can be taken from mean of **Y** from the RCM in both x and y directions respectively. *D*_*i*_ and *𝜃*_*i*_ are distances from the mean and angle from the x-axis, respectively. We have *n* coordinate pairs and for each case, *𝜃*_*i*_ can be given as:

8 $$ \theta_{i} = (i-1)\frac{2\pi}{n}  $$

In the first iteration, we can calculate the force at the contour point defined as follows:

9 $$ F_{D_{i}}=\frac{1}{N_{D_{i}}} {\sum}_{\theta_{i}-\frac{2\pi}{N_{D_{i}}}\leq \theta_{i} \leq \theta_{i}+\frac{2\pi}{N_{D_{i}}}} \textbf{F}(x_{i},y_{i}).[cos \theta_{i}, sin \theta_{i}]  $$

where $N_{D_{i}}$ is the number of neighbourhood points lying between the range [$\theta _{i}-\frac {2\pi }{N_{D_{i}}}$ $\theta _{i}+\frac {2\pi }{N_{D_{i}}}$]. The idea is to determine the force field including the contour point and its neighbour so as to avoid false edges due to occlusion. After determining the force field, the distances of the contour from the centre can be updated as:

10 $$\begin{array}{@{}rcl@{}} D_{inew}&=&D_{i} + \delta D, \text{ if } F_{Di}\geq t_{D} \\ D_{inew}&=&D_{i} - \delta D, \text{ if } F_{Di}\leq -t_{D} \end{array} $$

where *t*_*D*_ is the threshold which can be determined empirically based on the average force field of each contour point in the training set. Like the distance, the centre of the contour will also be updated. This can be achieved by defining the contour force in horizontal (*F*_*ch*_) and vertical (*F*_*cv*_) directions. We can define these forces as:

11 $$\begin{array}{@{}rcl@{}} F_{ch}=\frac{1}{n}\sum\limits_{i = 1}^{n}(\textbf{Y}{x_{i}},\textbf{Y}{y_{i}}).[1,0]^{T} \\ F_{cv}=\frac{1}{n}\sum\limits_{i = 1}^{n}(\textbf{Y}{x_{i}},\textbf{Y}{y_{i}}).[0,1]^{T} \end{array} $$

The contour centre can be updated as:

12 $$ \begin{array}{l} \left\{\begin{array}{l} x_{cnew}=x_{c}+\delta x_{c}, \text{ if } F_{ch}\geq t_{c} \\ x_{cnew}=x_{c}-\delta x_{c}, \text{ if } F_{ch}\leq -t_{c} \end{array}\right.\\ \left\{\begin{array}{l} y_{cnew}=y_{c}+\delta y_{c}, \text{ if } F_{cv}\geq t_{c} \\ y_{cnew}=y_{c}-\delta y_{c}, \text{ if } F_{cv}\leq -t_{c} \end{array}\right. \end{array} $$

where *t*_*c*_ is the threshold which can be determined empirically based on minimum centre force field in the training set. The contour is updated as:

13 $$ \begin{array}{l} \textbf{Y}{x_{inew}}=x_{cnew}+D_{inew}cos\theta_{i} \\ \textbf{Y}{y_{inew}}=y_{cnew}+D_{inew}cos\theta_{i} \end{array}  $$

**Fig. 6:**
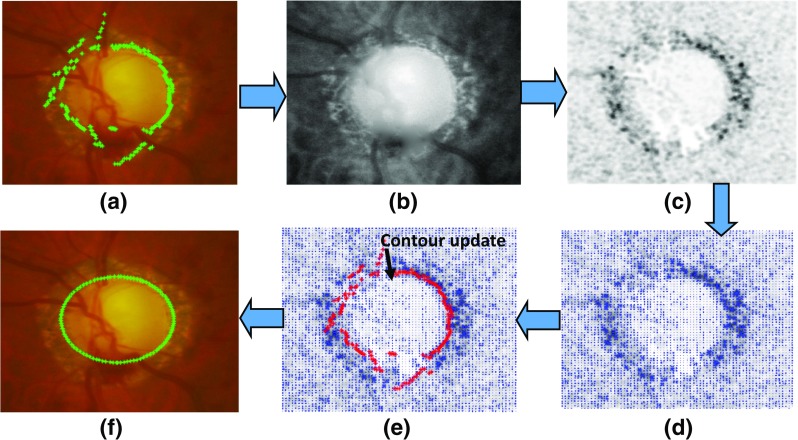
The procedure of adaptive edge smoothing update with **a** optimum contour from the RCM, **b** best feature map for determination of optic disc edge, **c** Edge map after convolving b with DoG filter, **d** force field of (**c**), **e** contour update towards maximum force and **f** final disc contour

This iterative process continues until convergence, i.e. **F**(**Y** *x*_*new*_,**Y** *y*_*new*_) −∇*I* ≈ 0. The update process is illustrated in Fig. 6.

### ARESM application to segmentation of optic disc and cup

#### Optic disc segmentation

**Fig. 7:**
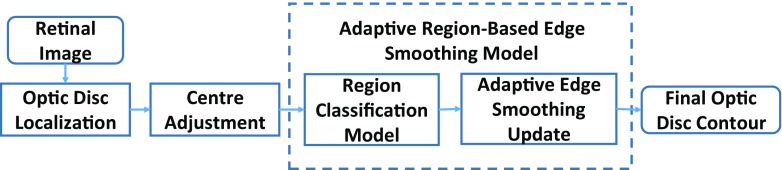
The overview of optic disc segmentation based on the proposed approach

Due to differences between the optic disc and optic cup, we have applied our proposed approach with a certain customisation for segmentation of the optic disc and cup, respectively. Optic disc localisation is the first step towards accurate segmentation of optic disc and cup. We have described our optic disc localisation method in [40]. It is based on enhancing both the optic disc and retinal vasculature convergence point whilst avoiding bright lesions and instrument reflections. The method is capable of locating the point somewhere within the optic disc boundary. However, the region initialisation can be more accurate if it is located closer to the optic disc centre. The optic disc centre can be readjusted to the centroid of the overlap area of the vasculature structure and the optic disc segmented within the area twice the size of optic disc. The vasculature can be segmented as mentioned in [45] whereas the optic disc can be segmented by binary classification from the RCM. The overview of optic disc segmentation is shown in Fig. 7. Furthermore, the optimum contour obtained from the RCM needs to be updated according to the AESU model which updates the contour according to the force field direction in every step by minimising energy function. To reduce undesirable influence of vasculature obscuration or PPA, and enhance the region of interest, we calculate the external energy of the image as the convolution between the image and the DoG filter which is the difference between two Gaussian filters defined at different scales, i.e. $-{\Gamma }_{\sigma _{1},\sigma _{2}}(x,y)*I(x,y)$, where ${\Gamma }_{\sigma _{1},\sigma _{2}}(x,y)$ as defined in Table 1.

#### Optic cup segmentation

Compared to the optic disc segmentation, the optic cup segmentation is a more challenging task for two reasons: a) there is no clear or distinct boundary between optic cup and rim of optic disc and b) vasculature occlusion. We have adapted our proposed approach to optic cup segmentation in two ways: a) the use of the prior knowledge in the RCM for accurate detection of optic cup by adding an additional feature, namely distance maps; b) adaptive smoothing update using the weighted features to make the optic cup force field more influential and enhance the region of interest (ROI) by performing multiplication between the external energy function and the weighted features from the RCM.

**Fig. 8:**
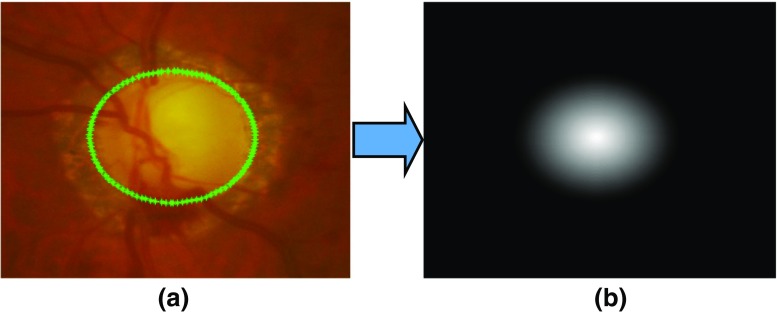
Determination of distance map (**b**) after optic disc segmentation as mentioned (**a**). The distance map shows higher pixel values near the centre indicating the higher chances of the pixel to be the part of optic cup

Normally, the optic cup has higher vasculature occlusion since the vascular tree converges towards the centre of the optic cup. This occlusion can affect the brighter portion of optic cup, which can distinguish between optic disc rim and optic cup, especially in case of normal images. Therefore we have introduced the *Distance Map* as an additional feature (the prior knowledge) to the classification model (RCM) between the optic disc rim and the optic cup. The distance map can be determined by the Eq. 14:

14 $$ \textbf{D}_{i}=\frac{1}{\sqrt{(\textbf{Y}{x_{inew}}-x_{cnew})^{2}+(\textbf{Y}{y_{inew}}-y_{cnew})^{2})}}  $$

where **Y** *x*_*inew*_, **Y** *y*_*inew*_ are updated contour points by AESU from Eq. 13 and *x*_*cnew*_, *y*_*cnew*_ are the updated centre. The concept behind adding the distance map is based on the fact that there is a higher probability of optic cup if the pixel lies near the centre of the optic disc. Therefore, the prior values for the pixel near the optic disc centre would be higher than the those away from the centre. Optic cup segmentation is performed after optic disc segmentation. Therefore the feature training will be performed between the optic disc rim and the optic cup. As an example, the distance map for the optic cup is shown in Fig. 8.

**Fig. 9:**
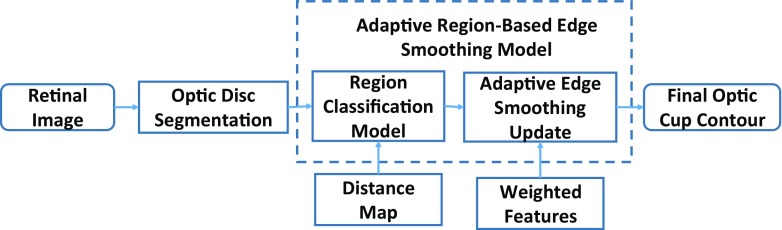
The overview of optic cup segmentation based on the proposed approach

Since the gradient is a very poor approximation of the boundary between the rim and the cup, we have redefined the external energy function by multiplying the weighted features obtained from the RCM. The purpose of performing the multiplication is to enhance the gradient for determining optic cup boundary. The modified external energy function for the optic cup is $-{\Gamma }_{\sigma _{1},\sigma _{2}}(x,y)*I(x,y).*net_{c}$, where . ∗ is dot multiplication, *I*(*x*,*y*) is the feature map best suited for optic cup segmentation and *n* *e* *t*_*c*_ is weighted features based on logistic output of the ANN. The workflow of the optic cup segmentation is shown in Fig. 9.

## Experimental evaluation

### Evaluation metrics

The optic disc and cup segmentation results have been compared with the masks obtained from the clinical annotations. The ***Dice Coefficient*** is used to determine the degree of overlap of the mask from clinical annotation and segmentation results and for determining the extent to which the segmented objects match [32, 46], defined as follows:

15 $$ D(A,B) = \frac {2 |A \cap B|}{|A| + |B|},  $$

where *A* and *B* are the segmented regions surrounded by model boundary and annotations from the ophthalmologists respectively, ∩ represents intersection. Its value varies between 0 and 1. A higher value means an increased degree of overlap. For optic cup segmentation accuracy, it is dependent on accurate optic disc segmentation as defined as follows:

16 $$ D_{oc} = \frac{2(|Rim_{1} \cap Rim_{2}| + |OC_{1} \cap OC_{2}|)}{N_{tot}}  $$

where *R* *i* *m*_1_ and *R* *i* *m*_2_ are the rim pixels and *O* *C*_1_ and *O* *C*_2_ are the optic cup pixels obtained from the benchmark and the automatic segmentation respectively, *N*_*tot*_ = *R* *i* *m*_1_ + *R* *i* *m*_2_ + *O* *C*_1_ + *O* *C*_2_.

The performance of our approach has also been evaluated by determining mean absolute difference between a clinical CDR and a CDR from the automatic segmentation results. The **absolute difference** can be given as:

17 $$ \delta = |CDR_{c} - CDR_{s}|  $$

where *C* *D* *R*_*c*_ is CDR from clinical annotations and *C* *D* *R*_*s*_ is the CDR from segmentation results. The CDR values have been evaluated in vertical, horizontal meridian as well as ratio of area covered by optic cup to the area covered by optic disc.

### Datasets

To validate the proposed approach, we have used two publicly available datasets: RIM-ONE [47] and Drishti-GS datasets [48]. The datasets used in the experiments include retinal images consisting of normal, glaucoma and glaucoma suspect images. The number of images in total is 209.

**RIM-ONE** (An Open Retinal Image Database for Optic Nerve Evaluation) [47] is a fundus image dataset. The latest release is composed of 85 normal, 39 glaucoma and 35 glaucoma suspect images; 159 in total with dimensions of 1072 × 1424 pixels. All the images have been annotated by two experts with boundaries of optic disc and optic cup. The interobserver variability of the RIM-ONE dataset has been calculated by the Dice Coefficients (“Evaluation metrics”) as shown in Table 2. There is 5% difference for optic disc and 7% difference for optic cup with 2% standard deviation on average amongst different annotations from the experts. Therefore, we evaluated the mean of annotations of both experts for each optic disc and cup in each image. An averaged outline of the annotations of all experts was obtained by taking, at each angle, the mean distance to the annotations from the mean centroid. The retinal images were obtained from three different hospitals across different regions of Spain to ensure a collection of representative and heterogeneous dataset. All images are non-mydriatic retinal photographs, which were collected with specific flash intensities to avoid saturation. This dataset has been used for the training and testing.

**Table 2:** Inter-observer variability in the datasets

RIMONE (1 vs 2)			Drishti-GS		
Image Type	Optic Disc	Optic Cup	Expert X vs Expert Y	Optic disc	Optic cup
Normal images	4.5% ± 2.07%	6.93% ± 2.22%	1 vs 2	1.00% ± 0.39%	1.47% ± 0.83%
Glaucoma images	5.01% ± 3.15%	7.31% ± 3.81%	1 vs 3	1.87% ± 0.61%	3.07% ± 1.57%
All images	4.74% ± 2.63%	7.11% ± 3.06%	1 vs 4	2.99% ± 1.35%	5.31% ± 2.10%
			2 vs 3	0.84% ± 0.27%	1.57% ± 0.94%
			2 vs 4	1.96% ± 1.20%	3.81% ± 1.61%
			3 vs 4	1.09% ± 1.02%	2.22% ± 1.25%

**Drishti-GS** dataset contains 50 images [48] in total. All images were captured at Aravind Eye Hospital, India. The patients were selected based on the age (ranging from 40 to 80 years old) and the gender (roughly equal number of males and females). The images taken were centred around optic disc with a field-of-view(FOV) of 30-degrees and of dimensions 2896 × 1944 pixels and PNG uncompressed image format. Four experts with various clinical experiences of 3, 5, 9 and 20 years annotated optic disc and optic cup of the images (Table 2). Drishti dataset does not specify if the image is glaucomatous or not. However, its average CDR values are comparatively higher than those in RIM-ONE (Table 3). This dataset has been used for the testing purpose only.

**Table 3:** Average CDR values (vertical, horizontal and area) in the RIMONE and Drishti datasets

	RIMONE			Drishti-GS
CDR Type	Normal	Glaucoma	Both	
Vertical	0.42 ± 0.10	0.60 ± 0.17	0.50 ± 0.16	0.69 ± 0.13
Horizontal	0.40 ± 0.11	0.57 ± 0.16	0.48 ± 0.16	0.70 ± 0.14
Area	0.18 ± 0.09	0.37 ± 0.19	0.27 ± 0.17	0.51 ± 0.18

### Model parameterisation

The ARESM requires parameterisation for accurate boundary detection and segmentation, which are related to several factors: 1) whether vasculature removal is required before feature selection; 2) feature selection; 3) training protocol and 4) classifier and contour profile optimisation parameters. The following sections details the approach of model parameterisation.

#### Determination on whether vasculature removal is required

**Fig. 10:**
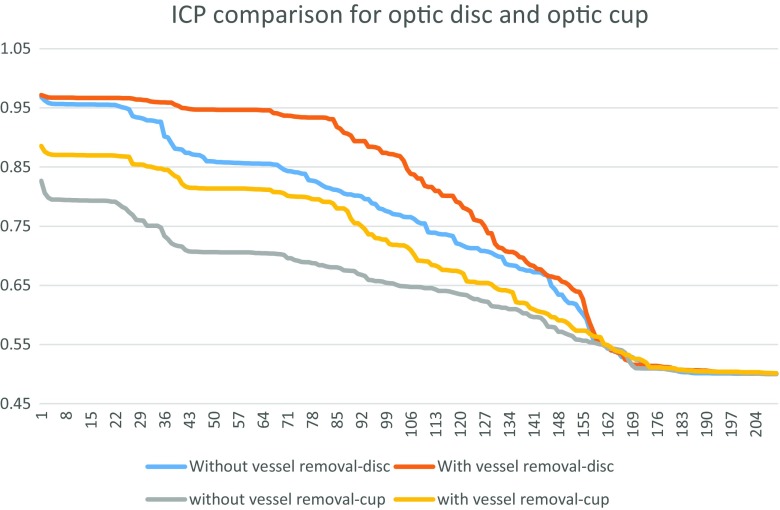
Comparison of the individual classification performance with and without vasculature removal for optic disc and optic cup. The result shows that the vasculature removal has higher individual classification performance

For the determination of feature sets for the RCMs of the optic disc and optic cup, we have the features determined with and without vasculature removal. The results of the features with highest Individual Classification Performance (ICP) (which is the measure of individual performance of each feature in terms of classification power) for both cases with and without vasculature removal is shown in Fig. 10.

The ICPs have been measured in terms of AUC value for individual features. The results show that for both optic disc and optic cup, the features calculated with vasculature removal have higher ICPs compared to those calculated with and without vasculature removal. Therefore we have determined the feature set on those after vasculature removal.

#### Feature selection

**Fig. 11:**
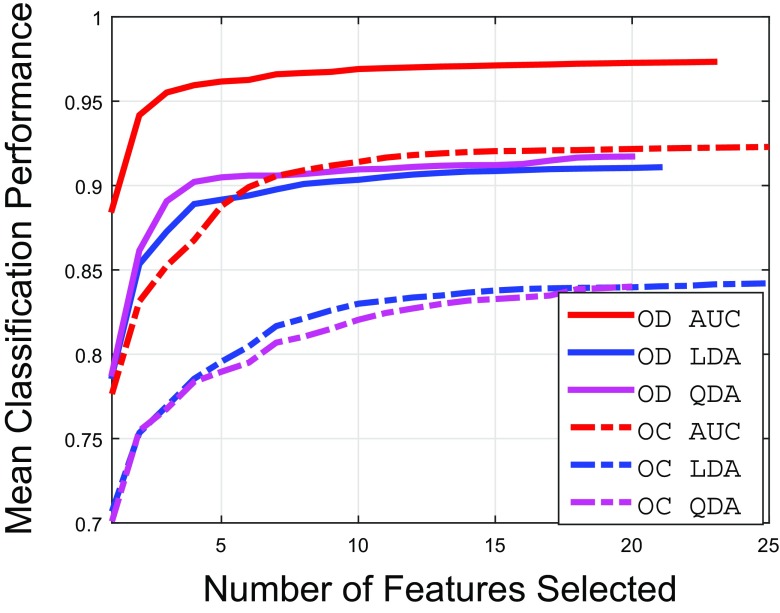
Results of feature selection procedures for optic disc (OD) and optic cup (OC)

The results of the feature selection procedure with different performance measures (as discussed in “Feature extraction and selection”) are shown in Fig. 11. The results show that if the features are selected by AUC as performance measure of sequential maximisation, we can achieve significantly higher classification accuracy compared to other performance measures. The list of selected features in Table 4 (also represent x-axis of Fig. 11 for features selected by AUC sequential maximisation) show that the Gaussian derived features dominate the feature set for the optic disc extraction since they have strong edge information at different scales. The feature sets selected for optic cup extraction are mostly dominated by Gaussian features and Dyadic Gaussian Features.

**Table 4:** Feature symbols for each feature set obtained by sequential AUC maximisation for optic disc and optic cup region determination

Optic disc	Optic cup
$\mathcal {N}_{R}(16)$, *I*_*mn*_(4, 8),	$\mathcal {N}_{R}(16)$, $ \mathcal {N}_{xxR}(16)$,
*B* *Y* (4, 8), *L*_*u*,*uG*_(8),	Γ_16,4,*G*_, *L*_*u*,*uG*_(8),
$\mathcal {N}_{G}(16)$, *L*_*uuG*_(16),	*B* *Y* (4, 7),*I*_*mn*_(4, 8),
*L*_*qq*,*γ*−*normR*_(16),	*L*_*uv*,*vuG*_(16), $\mathcal {N}_{G}(16)$,
*I*_*mn*_(4, 7), *B* *Y* (4, 7),	*I*_*mn*_(4, 7), *I*_*mn*_(3, 7), $\mathcal {N}_{R}(8)$,
*L*_*uvR*_(4), *L*_*uv*,*vuG*_(2),	$\mathcal {N}_{G}(16)$, $\mathcal {N}_{yG}(8)$,
*L*_*uuG*_(2), *L*_*uvG*_(4),	$\mathcal {N}_{yyG}(16)$, $\mathcal {N}_{xxR}(8)$,
*L*_*uuR*_(16), *L*_*uv*,*vuR*_(16),	*L*_*qq*,*γ*−*normG*_(8),
*L*_*uv*,*vuG*_(16), Γ_8,4,*R*_,	*R* *G*(4, 8), $\mathcal {N}_{xxG}(16)$,
*L*_*qq*,*γ*−*normG*_(16), Γ_4,2,*G*_,	Γ_16,4,*G*_
*L*_*uv*,*vuR*_(8)	

#### Training protocol

The training was performed on the RIMONE dataset with 2-fold cross validation i.e. training one part whilst testing the other. The Drishti dataset has been tested on the model built upon the RIMONE dataset.

**Fig. 12:**
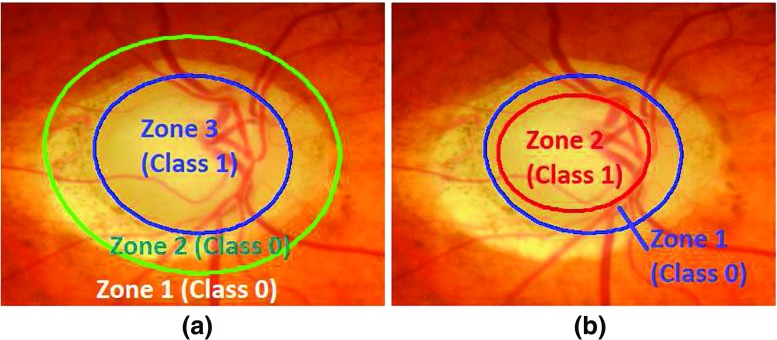
Classification zones for **a** optic disc and **b** optic cup. The classification of optic disc has been performed between inside and outside of optic disc whereas classification for optic cup has been performed between inside of optic cup and optic disc rim

As far as the training is concerned, we have a binary classification problem for generating RCMs for both optic disc and optic cup. The pixel-wise training can make the training set very large which can slow down the training process. Also for the optic disc, we need to train the features which are part of the atrophy region as well as the retinal area. Therefore, we have divided optic disc into 3 zones in which zone-1 and zone-2 belong to class-0 (outside optic disc) and zone-3 belong to class-1 (inside optic disc) (Fig. 12). Zone-1 belongs to retinal area whereas zone-2 belongs to the atrophy region. We have randomly selected 2000 samples from zone-3 and 1000 samples from each zone-1 and zone-2 of each optic disc cropped image in the training set. For optic cup, the procedure is the same except that we have removed vasculature area after segmenting out and morphological closing [45] as well as the training has been performed between the optic cup and the rim inside optic disc.

As far as training parameters are concerned in “Contour profile optimisation”, we have trained a single layer backpropagation neural networks with the number of input and hidden neurons equal to the number of selected features (20 for optic disc and 25 for optic cup). However, the hidden neurons vary from 15 to 30 which will give a similar result. Other parameters include *n*_*p*_ = 25 − 35, *m* = 7 − 10, *n* = 200 (optic disc), 100 (optic cup).

### Accuracy comparison with state-of-the-art approaches

The optic disc and cup segmentation accuracy were determined on the basis of the following aspects:

- Accuracy performance comparison with the previous approaches.
- Accuracy performance comparison based on CDR values (Cup-Disc-Ratio).

#### Optic Disc Segmentation Accuracy Comparison

**Fig. 13:**
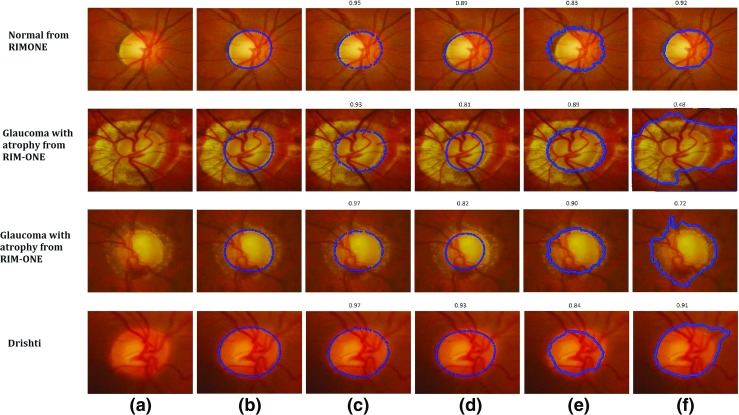
Examples of Optic Disc Segmentation Results with **a** Original Image **b** Clinical Annotations, **c** ARESM (our proposed approach), **d** ASM, **e** ACM and **f** Chan-Vese (C-V). The Dice Coefficient of each method compared to ground truth has been shown above each visual result

We applied our approach to the datasets above and compared it with existing approaches including ASM [29–31], ACM [19, 49] and Chan-Vese (C-V) [22, 50] models. We used the mean Dice Coefficient ***D*** to measure the accuracy of ***the optic disc segmentation*** across different datasets and methods as shown in Table 5. Some examples of optic disc segmentation results based on different methods are shown in Fig. 13. The second and third rows in Fig. 13 present the optic disc boundary segmentation by the different methods in the presence of PPA.

**Table 5:** Comparison of optic disc segmentation results - mean and standard deviations of dice coefficient

	RIMONE			Drishti-GS
	Normal	Glaucoma	All	
ARESM	0.92 ± 0.06	0.90 ± 0.07	0.91 ± 0.07	0.95 ± 0.02
ASM model	0.85 ± 0.10	0.77 ± 0.16	0.76 ± 0.13	0.87 ± 0.06
ACM model	0.86 ± 0.07	0.85 ± 0.09	0.86 ± 0.08	0.91 ± 0.03
C-V model	0.88 ± 0.13	0.86 ± 0.14	0.87 ± 0.14	0.85 ± 0.11

Based on the results, our proposed approach yield the highest Dice Coefficients in comparison with the existing models. Figure 13c shows the examples of visual results. The ASM model-based segmentation is misguided as the mean of the shape in the training set keeps constant (once it is trained). Figure 13d shows the examples of visual results. The ACM and C-V model-based segmentations are comparable to our approach on these images. Nevertheless their performance is uncertain as presented in other examples. The ACM is dependent on the strong edges of the optic disc which might not be the case in every example. The examples of visual results are shown in Fig. 13e. Optic disc margin segmentation errors can occur using the Chan Vese model when PPA is present (Fig. 13f). For RIM-One dataset, the average accuracy of optic disc segmentation is 91%. The accuracy of Drishti-GS is 95%.

#### Optic cup segmentation accuracy comparison

**Fig. 14:**
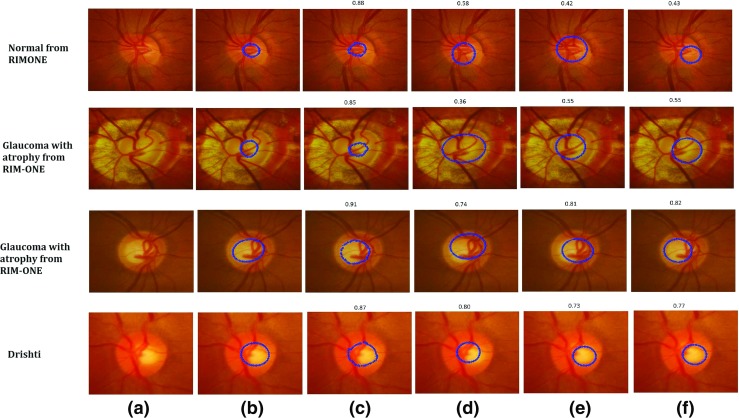
Examples of Optic Cup Segmentation Results with **a** Original Image **b** Clinical Annotations, **c** ARESM (our proposed approach), **d** ASM, **e** ACM and **f** Chan-Vese (C-V). The Dice Coefficient of each method compared to ground truth has been shown above each visual result

Compared to optic disc segmentation, optic cup segmentation is more challenging as there is no clear or distinct boundary of the optic cup. The cup has a volume and the image is really a 2d presentation of a 3D structure. Moreover, in the normal optic nerve the cup is typically smaller and it’s margins are often obscured by retinal vessels. Previous methods such as the Fuzzy C-means (FCM) clustering [51] and thresholding [52] often fail to correctly outline the optic cup boundary. Since the proposed approach is based on the deformable model approach, we have compared our method with existing deformable approaches (ASM, ACM, C-V). The performance of our proposed approach produces the highest Dice Coefficients compared to these approaches in both large and small size optic cups as shown in Table 6. Some examples of optic cup segmentation results are shown in Fig. 14.

**Table 6:** Comparison of optic cup segmentation results - mean and standard deviations of dice coefficient

	RIMONE			Drishti-GS
	Normal	Glaucoma	All	
ARESM	0.91 ± 0.06	0.89 ± 0.06	0.89 ± 0.06	0.81 ± 0.10
ASM	0.78 ± 0.09	0.73 ± 0.13	0.76 ± 0.12	0.72 ± 0.14
ACM	0.76 ± 0.10	0.81 ± 0.09	0.79 ± 0.10	0.71 ± 0.12
C-V	0.71 ± 0.18	0.73 ± 0.17	0.72 ± 0.18	0.80 ± 0.08

The results suggest that the proposed method has achieved high accuracy, in comparing with the existing approaches. The reason is that optic cup segmentation has been highly dependent on the accurate optic disc segmentation. Moreover, the algorithms like ACM and C-V are not converged in the case of cup segmentation. The ASM produces annotation failures due to vasculature occlusion which is not the case in the ARESM. The ARESM method does fail in some of the normal images due to their very small size of optic cup. Nevertheless, the cup segmentation has an average accuracy of 89% for RIM-ONE and 81% for Drishti-GS database images.

#### Accuracy Comparison based on CDR

CDR is an important indicators related to glaucoma diagnosis. Accurate automated CDR assessment requires precise assessment of the cup and disc margins compared to an acceptable reference standard.

In order to compare the clinical manual CDR and automatically determined CDR, we have used the RIM-ONE dataset in which both the optic disc and optic cup have been manually annotated by clinicians. Hence the manual CDR values can be calculated from these two datasets in the a) vertical meridian, b) horizontal meridian; and c) area. However, CDR in the vertical meridian is used most commonly by clinicians in optic nerve evaluation for glaucoma [1].

Both datasets contain three types of images: *normal*, *glaucoma* and *glaucoma suspect* images. Therefore, we have evaluated the CDRs (i.e. vertical CDR, horizontal CDR and area CDR) in two sets 1) normal (N) vs glaucoma (G) and 2) normal (N) vs glaucoma and suspects (G + S). The RIM-ONE dataset has 85 normal (N), 39 glaucoma (G) and 35 glaucoma suspect (S) images.

**Fig. 15:**
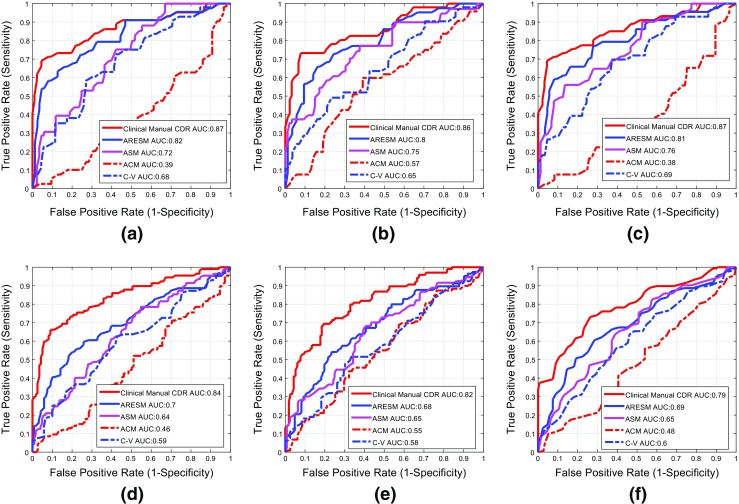
Comparison of classification performance between the clinical manual CDR and the automatic CDRs with the first row **a**, **b** and **c** represents the results on set 1 (N vs G) and second row represents the results on set 2 (N vs (G + S)). The first column ((**a**) and (**d**)) represent the results calculated on vertical CDR whereas the second and third column represent the results on horizontal CDR and the area CDR respectively

Receiver Operating Characteristic (ROC) curves have been generated to illustrate the classification performance between the manual CDRs based on clinical and automated classifications as shown in Fig. 15. The paired t-test has been used to compare the ROC curves generated by the manual CDRs with those obtained from automatic CDRs. The difference between the ROC curves were declared to be significant if the *p*-value is less than 0.05 [53]. This determines confidence level of using the automatic CDRs clinically. The first row in Fig. 15 shows ROC curves about classification performance between the manual and automatic CDRs on the first set (normal (N) and glaucoma (G)). The second row shows the ROC curves about classification between the manual and the automatic CDRs on normal and glaucoma plus glaucoma suspect (G+S) images. Table 7 shows the *p*-values calculated by the paired t-test between ROC curves generated by the automatic CDRs and the manual CDRs. The results show that there is no significant difference between the ROC curves generated by the manual CDRs and the ROC curves generated by ARESM CDRs as far as classification between Normal and Glaucoma is concerned. This demonstrates that if the CDR is considered as one of the clinical measurement for glaucoma classification, the CDRs generated by the ARESM can be used to faciltate automatic classification between normal and glaucoma. However, it needs to be tested on the large set of images as there are other clinical factors which need to be considered.

**Table 7:** Comparison between our proposed approach (ARESM), the clinical manual CDR and the other existing methods in terms of **mean CDR error** and ***p*** *-values* of the paired t-test which shows the comparison between ROC curves generated by manual CDRs and the CDRs from automatic methods

	Vertical CDR				Horizontal CDR						Area CDR				
	N	G	G+S	All	*p*-value (N vs. G)	N	G	G+S	All	*p*-value (N vs. G)	N	G	G+S	All	*p*-value (N vs. G)
ARESM	0.08	0.11	0.08	0.07	0.05	0.08	0.10	0.08	0.07	0.16	0.05	0.11	0.08	0.06	0.02
ASM	0.22	0.21	0.21	0.22	< 0.0001	0.31	0.29	0.27	0.29	0.05	0.26	0.33	0.29	0.27	< 0.0001
ACM	0.20	0.13	0.13	0.17	< 0.0001	0.20	0.12	0.12	0.16	< 0.0001	0.19	0.14	0.13	0.16	< 0.0001
C-V	0.13	0.14	0.14	0.13	< 0.0001	0.12	0.13	0.12	0.12	< 0.0001	0.09	0.14	0.12	0.11	< 0.0001

The CDR values are generated in vertical meridian, horizontal meridian and area ratio of cup and disc. *p*-values are generated by comparing ROC curves in terms of normal (N) vs. glaucoma(G)

## Conclusion

In this work, we have presented a novel solution to accurately segment the optic disc and optic cup. In contrast to the existing approaches, the novelty of the proposed approach lies in two aspects:

- We have developed the Region Classification Model (RCM) which identifies the initial optimum contour approximation representing optic disc or cup boundary between inside and outside region of interest based on pixel-wise classification in a multidimensional feature space, and performs region search for optimum contour profile. This is different from the existing models such as the conventional ASM model where the contour is static once it has been trained from the training set. Our model can dynamically search the region and obtain the most optimum contour.
- To overcome misclassification and irregularity of contour points, we have proposed the Adaptive Edge Smoothing Update model (AESU) which can dynamically smooth and update the irregularities and misclassified points by minimising the energy function according to the force field direction in an iterative manner. Our model does not require a predefined template such as a circle or an ellipse. It could be any contour generated from the RCM model. This is different from the existing approaches which used a circular or ellipse fitting for smoothing update.

We have applied our approach to both optic disc and optic cup segmentations. We have conducted a comprehensive comparison with the existing approaches such as ASM, ACM, and Chan-Vese (C-V) models. The approaches were validated with two publicly available data sets: RIM-ONE and Drishti-GS.

For *optic disc segmentation* of RIM-One dataset, the average accuracy of optic disc segmentation is 91%. The accuracy of Drishti-GS is 95%. It should be noted that the proposed approach works well on high resolution images of RIM-ONE since the low resolution images have blurred vasculature and optic disc edges. For *optic cup* segmentation, the average accuracy of optic cup segmentation is 89% for RIM-ONE and 81% for Drishti-GS databases. The optic cup segmentation results are highly dependent on the accurate segmentation of the optic disc segmentation. Moreover, failed cases of optic cup segmentation include the normal images which have very small cup size. Future work will focus on more accurate segmentation of the small cup.

Based on the rationale outlined here, our proposed approach can also be applied to boundary detection of other objects. Future work is needed to apply this algorithm to other object boundary detections and also improve accuracy of the ARESM model.

## References

[1] Jonas J., Budde W., Jonas S.: Ophthalmoscopic evaluation of optic nerve head. Surv. Ophthalmol. 43: 293–320, 1999 10.1016/s0039-6257(98)00049-6 10025513

[2] Tangelder G., Reus N., Lemij H.: Estimating the clinical usefulness of optic disc biometry for detecting glaucomatous change over time. Eye 20: 755–763, 2006 10.1038/sj.eye.6701993 15999126

[3] Liu J., Lim J. H., Wong W. K., Li H., Wong T.Y. (2011) Automatic cup to disc ratio measurement system. http://www.faqs.org/patents/app/20110091083

[4] Haleem M. S., Han L., van Hemert J., Li B.: Automatic extraction of retinal features from colour retinal images for glaucoma diagnosis: a review. Comput. Med. Imaging Graph. 37: 581–596, 2013 10.1016/j.compmedimag.2013.09.005 24139134

[5] Mahapatra D.: Combining multiple expert annotations using semi-supervised learning and graph cuts for medical image segmentation. Comput. Vis. Image Underst. 151: 114–123, 2016. Probabilistic Models for Biomedical Image Analysis

[6] Qureshi R. J., Kovacs L., Harangi B., Nagy B., Peto T., Hajdu A.: Combining algorithms for automatic detection of optic disc and macula in fundus images. Comput. Vis. Image Underst. 116(1): 138–145, 2012

[7] Salazar-Gonzalez A., Kaba D., Li Y., Liu X.: Segmentation of the blood vessels and optic disk in retinal images. IEEE Journal of Biomedical and Health Informatics 18(6): 1874–1886, 2014 10.1109/JBHI.2014.2302749 25265617

[8] Zhang D., Zhao Y.: Novel accurate and fast optic disc detection in retinal images with vessel distribution and directional characteristics. IEEE Journal of Biomedical and Health Informatics 20(1): 333–342, 2016 10.1109/JBHI.2014.2365514 25361515

[9] Roychowdhury S., Koozekanani D. D., Kuchinka S. N., Parhi K. K.: Optic disc boundary and vessel origin segmentation of fundus images. IEEE Journal of Biomedical and Health Informatics 20(6): 1562–1574, 2016 10.1109/JBHI.2015.2473159 26316237

[10] Zhao Y., Zheng Y., Liu Y., Yang J., Zhao Y., Chen D., Wang Y.: Intensity and compactness enabled saliency estimation for leakage detection in diabetic and malarial retinopathy. IEEE Trans. Med. Imaging 36(1): 51–63, 2017 10.1109/TMI.2016.2593725 27455519

[11] Zhao Y., Liu Y., Wu X., Harding S. P., Zheng Y.: Retinal vessel segmentation: an efficient graph cut approach with retinex and local phase. PloS one 10(4): e0122332, 2015 10.1371/journal.pone.0122332 PMC4382050 25830353

[12] Nayak J., Acharya R., Bhat P., Shetty N., Lim T. -C.: Automated diagnosis of glaucoma using digital fundus images. J. Med. Syst. 33: 337–346, 2009 10.1007/s10916-008-9195-z 19827259

[13] Walter T., Klein J. -C.: Segmentation of color fundus images of the human retina: detection of the optic disc and the vascular tree using morphological techniques.. In: Proceedings of the Second International Symposium on Medical Data Analysis, 2001, pp 282–287

[14] Vishnuvarthanan A., Rajasekaran M. P., Govindaraj V., Zhang Y., Thiyagarajan A.: An automated hybrid approach using clustering and nature inspired optimization technique for improved tumor and tissue segmentation in magnetic resonance brain images. Appl. Soft Comput. 57: 399–426, 2017

[15] Wang S., Li Y., Shao Y., Cattani C., Zhang Y., Du S.: Detection of dendritic spines using wavelet packet entropy and fuzzy support vector machine. CNS Neurol. Disord. Drug Targets (Formerly Curr. Drug Targets CNS Neurol. Disord.) 16(2): 116–121, 2017 10.2174/1871527315666161111123638 27834129

[16] Abdel-Ghafar R., Morris T.: Progress towards automated detection and characterization of the optic disc in glaucoma and diabetic retinopathy. Inform. Health Soc. Care 32(1): 19–25, 2007 10.1080/14639230601095865 17365641

[17] Lalonde M., Beaulieu M., Gagnon L.: Fast and robust optic disc detection using pyramidal decomposition and hausdorff-based template matching. IEEE Trans. Med. Imaging 20: 1193–1200, 2001 10.1109/42.963823 11700746

[18] Pallawala P., Hsu W., Lee M., Eong K.: Automatic localization and contour detection of optic disc.. In: ECCV, 2004, pp 139–151

[19] Kass M., Witkin A., Terzopoulous D.: Snakes: active contour models. Int. J. Comput. Vis. 1(4): 321–331, 1987

[20] Zhao Y., Zhao J., Yang J., Liu Y., Zhao Y., Zheng Y., Xia L., Wang Y.: Saliency driven vasculature segmentation with infinite perimeter active contour model. Neurocomputing 259: 201–209, 2017

[21] Sethian J (1999). Level set methods and fast marching methods.

[22] Chan T., Vese L.: An active contour model without edges. IEEE Trans. Image Process. 10(2): 266–277, 2002 10.1109/83.902291 18249617

[23] Joshi G., Sivaswamy J., Krishnadas S.: Optic disk and cup segmentation from monocular color retinal images for glaucoma assessment. IEEE Trans. Med. Imaging 30: 1192–1205, 2011 10.1109/TMI.2011.2106509 21536531

[24] Lowell J., Hunter A., Steel D., Basu A., Ryder R., Fletcher E., Kennedy L.: Optic nerve head segmentation. IEEE Trans. Biomed. Eng. 23: 256–264, 2004 10.1109/TMI.2003.823261 14964569

[25] Xu J., Chutatape O., Sung E., Zheng C., Kuan P. C. T.: Optic disk feature extraction via modified deformable model technique for glaucoma analysis. Pattern Recogn. 40: 2063–2076, 2007

[26] Wong D., Liu J., Lim J., Jia X., Yin F., Li H., Wong T.: Level-set based automatic cup-to-disc ratio determination using retinal fundus images in argali.. In: 30th Annual International Conference of the IEEE Engineering in Medicine and Biology Society, 2008, pp 2266–2269 10.1109/IEMBS.2008.4649648 19163151

[27] Osareh A., Mirmehdi M., Thomas B., Markham R.: Comparison of colour spaces for optic disc localisation in retinal images.. In: Proceedings of the 16th International Conference on Pattern Recognition, 2002, pp 743–746

[28] Tang Y., Li X., von Freyberg A., Goch G.: Automatic segmentation of the papilla in a fundus image based on the c-v model and a shape restraint.. In: Proceedings of 18th International Conference on Pattern Recognition (ICPR’06), vol 1, 2006, pp 183–186

[29] Cootes T., Taylor C. (2004) *Statistical models of appearance for computer vision*. Tech. Rep., University of Manchester

[30] Cheng J., Liu J., Yin F., Lee B. -H., Wong D. W. K., Aung T., Cheng C. -Y., Wong T. Y.: Self-assessment for optic disc segmentation.. In: Engineering in Medicine and Biology Society (EMBC), 2013 35th Annual International Conference of the IEEE. IEEE, 2013, pp 5861–5864 10.1109/EMBC.2013.6610885 24111072

[31] Yin F., Liu J., Ong S. H., Sun Y., Wong D. W., Tan N. M., Cheung C., Baskaran M., Aung T., Wong T. Y.: Model-based optic nerve head segmentation on retinal fundus images.. In: Engineering in Medicine and Biology Society, EMBC, 2011 Annual International Conference of the IEEE. IEEE, 2011, pp 2626–2629 10.1109/IEMBS.2011.6090724 22254880

[32] Fengshou Y. (2011) *Extraction of features from fundus images for glaucoma assessment*. Master’s Thesis, National University of Singapore

[33] Haleem M. S., Han L., Li B., Nisbet A., van Hemert J., Verhoek M.: Automatic extraction of optic disc boundary for detecting retinal diseases.. In: 14th IASTED International Conference on Computer Graphics and Imaging (CGIM), 2013, pp 40–47

[34] Li H., Chutatape O.: Boundary detection of optic disk by a modified asm method. Pattern Recogn. 36: 2093–2104, 2003

[35] Xu J., Chutatape O., Chew P.: Automated optic disk boundary detection by modified active contour model. IEEE Trans. Biomed. Eng. 54: 473–482, 2007 10.1109/TBME.2006.888831 17355059

[36] Abràmoff M., Alward W., Greenlee E., Shuba L., Kim C., Fingert J., Kwon Y.: Automated segmentation of the optic disc from stereo color photographs using physiologically plausible features. Investig. Ophthalmol. Vis. Sci. 48: 1665–1673 , 2007 10.1167/iovs.06-1081 PMC2739577 17389498

[37] Anderson C. H., Bergen J. R., Burt P. J., Ogden J. M.: Pyramid methods in image processing. RCA Engineer 29: 33–41, 1984

[38] Daugman J.: Complete discrete 2-d gabor transforms by neural networks for image analysis and compression. IEEE Trans. Acoust. Speech Signal Process. 36(7): 1169–1179, 1988

[39] Lindeberg T.: Edge detection and ridge detection with automatic scale selection. Int. J. Comput. Vis. 30(2): 117–154, 1998

[40] Haleem M. S., Han L., van Hemert J., Fleming A., Pasquale L. R., Silva P. S., Song B. J., Aiello L. P.: Regional image features model for automatic classification between normal and glaucoma in fundus and scanning laser ophthalmoscopy (slo) images. J. Med. Syst. 40(6): 132, 2016 10.1007/s10916-016-0482-9 PMC4834108 27086033

[41] Serrano A. J., Soria E., Martin J. D., Magdalena R., Gomez J.: Feature selection using roc curves on classification problems.. In: The International Joint Conference on Neural Networks (IJCNN), 2010, pp 1–6

[42] Smola A, Vishwanathan S (2008). Introduction to machine learning.

[43] Stegmann M., Gomez D. (2002) A brief introduction to statistical shape analysis. Tech. rep

[44] Yuan X., Giritharan B., Oh J.: Gradient vector flow driven active shape for image segmentation.. In: IEEE International Conference on Multimedia and Expo, 2007, pp 2058–2061

[45] Lupascu C. A., Tegolo D., Trucco E.: Fabc: retinal vessel segmentation using adaboost. IEEE Trans. Inf. Technol. Biomed. 14(5): 1267–1274, 2010 10.1109/TITB.2010.2052282 20529750

[46] Sun J., Luan F., Wu H. (2015) Optic disc segmentation by balloon snake with texture from color fundus image. *J. Biomed. Imaging* 4 10.1155/2015/528626 PMC4378594 25861249

[47] Fumero F., Sigut J., Alayón S., González-Hernández M., González M.: Interactive tool and database for optic disc and cup segmentation of stereo and monocular retinal fundus images.. In: Short Papers Proceedings–WSCG, 2015, pp 91–97

[48] Sivaswamy J., Krishnadas S. R., Joshi G.D., Jain M., Tabish A.U.S.: Drishti-gs: Retinal image dataset for optic nerve head (ONH) segmentation.. In: IEEE 11th International Symposium on Biomedical Imaging (ISBI). IEEE, 2014, pp 53–56

[49] Mary M. C. V. S., Rajsingh E. B., Jacob J. K. K., Anandhi D., Amato U., Selvan S. E.: An empirical study on optic disc segmentation using an active contour model. Biomed. Signal Process. Control 18: 19–29, 2015

[50] Joshi G. D., Sivaswamy J., Krishnadas S.: Optic disk and cup segmentation from monocular color retinal images for glaucoma assessment. IEEE Trans. Med. Imaging 30(6): 1192–1205, 2011 10.1109/TMI.2011.2106509 21536531

[51] Babu T. R. G., Shenbagadevi S.: Automatic detection of glaucoma using fundus image. Eur. J. Sci. Res. 59: 22–32, 2011

[52] Kose C., Ikibas C.: Statistical techniques for detection of optic disc and macula and parameters measurement in retinal fundus images. Journal of Medical and Biological Engineering 31: 395–404, 2010

[53] DeLong E., DeLong D. M., Clarke-Pearson D. L.: Comparing the areas under two or more correlated receiver operating characteristic curves: a nonparametric approach. Biometrics 44(3): 837–845, 1988 3203132

